# Photon antibunching in a cluster of giant CdSe/CdS nanocrystals

**DOI:** 10.1038/s41467-018-03971-w

**Published:** 2018-04-18

**Authors:** Bihu Lv, Huichao Zhang, Lipeng Wang, Chunfeng Zhang, Xiaoyong Wang, Jiayu Zhang, Min Xiao

**Affiliations:** 1 National Laboratory of Solid State Microstructures, School of Physics, and Collaborative Innovation Center of Advanced Microstructures, Nanjing University, 210093 Nanjing, China; 2 College of Electronics and Information, Hangzhou Dianzi University, Xiasha Campus, 310018 Hangzhou, China; 3 Advanced Photonics Center, School of Electronic Science and Engineering, Southeast University, 210096 Nanjing, China; 4 Department of Physics, University of Arkansas, Fayetteville, AR 72701 USA

## Abstract

When closely packed into a high-density film, semiconductor nanocrystals (NCs) can interact with each other to yield collective optical behaviours, which are normally difficult to characterize due to the ensemble average effect. Here we synthesized semiconductor NC clusters and performed single-particle spectroscopic measurements to probe the electronic couplings of several giant CdSe/CdS NCs contained in one cluster with nanometer-scale separations. Such a single cluster exhibits multiple emission peaks at the cryogenic temperature with nearly identical photoluminescence decay dynamics, suggesting that the Förster-type energy transfer does not occur among the composing NCs. Surprisingly, strong photon antibunching is still observed from a single cluster, which can be attributed to the Auger annihilation of photo-excited excitons from different NCs. The isolation of several nearby NCs interacting with the above novel mechanism has marked a solid progress towards a full understanding and an efficient control of the operation parameters in NC-based optoelectronic devices.

## Abstract (web summary)

Semiconductor nanocrystals have potential applications in optoelectronic devices including single-photon emission sources. Here, Lv et al. demonstrate that nanocrystals within small clusters can electronically couple, thereby increasing their optical cross-section while the optical emission remains antibunched.

## Introduction

Semiconductor nanocrystals (NCs) have been attracting a lot of research interests in both fundamental studies^1^ and practical applications^2^ owing to their size- and shape-dependent optical properties that are dictated by the quantum confinement effect. For the efficient usage in a variety of optoelectronic devices such as lasers, light-emitting diodes, photo-detectors, and solar cells, semiconductor NCs have to be closely packed into high-density films for the enhancements of the optical gain, the light absorption/emission and the charge transport capabilities. Consequently, it is imperative to have a comprehensive understanding of the collective behaviours resulting from the mutual interactions of neighbouring NCs, which mainly include the Förster energy transfer (ET) and coherent electronic coupling processes. The Förster ET from the donor to the acceptor NCs proceeds through the long-range dipole–dipole interaction whereby the photoluminescence (PL) intensity and lifetime of the donor NC is effectively reduced^3,4^. On the other hand, the short-range coherent coupling of nearby NCs is strongly governed by the spatial overlap of their electronic wave functions, leading to the broadening and red shift of the band-edge exciton transitions^5–7^. Limited by the extreme difficulty in isolating several adjacent NCs, there were only a few rare cases in the literature reporting single-particle spectroscopic measurements on the above two types of NC interactions to remove the ensemble average effect. For example, it was demonstrated from single clusters of colloidal CdSe/ZnS NCs that their PL blinking rate could be dramatically expedited^8^, which was suggested to be a direct consequence of the Förster ET^9^. Moreover, an apparent broadening of the conduction electron levels was previously revealed by the scanning tunneling spectroscopy as a strong indication of the delocalized electron wave functions within a cluster of colloidal PbSe NCs^10^. Very recently, the electron energy loss spectroscopy was applied to single perovskite CsPbBr_3_ NCs to demonstrate their band-structure modifications due to the presence of an effective coupling between proximal NCs in an ensemble^11^.

Colloidal giant CdSe/CdS NCs (gNCs) comprising a small CdSe core and a thick CdS shell have recently emerged as a novel type of light emitting/harvesting nanostructure^12,13^. They are associated with highly improved photostability, as well as superior optical properties, such as nonblinking PL^14^ and suppressed Auger recombination in both charged excitons^15^ and neutral multi-excitons^16^.

In the following, we show that a flower-like cluster can be effectively formed to contain about 4–5 CdSe/CdS gNCs with nanometer-scale separations by manipulating the synthesis conditions with the passivating ligands. Compared to individual gNCs at room temperature, such a single gNC cluster (gNCC) possesses a relatively larger absorption cross section, but otherwise exhibits very similar optical properties in terms of nonblinking PL, long single-exponential lifetime and strong photon antibunching effect. This quantum nature of single-photon emission is still kept for a single gNCC at the cryogenic temperature, although its broad PL spectrum measured at room temperature is now divided into multiple emission peaks with nearly identical PL decay dynamics to exclude the Förster ET interactions among the composing gNCs. We tentatively propose that the exciton recombination energy of one gNC could be nonradiatively transferred to the exciton confined in a nearby gNC through an inter-gNC Auger interaction process, which results in the high-purity single-photon emission from a single gNCC.

## Results

### Chemical synthesis of CdSe/CdS gNCCs

**Fig. 1:**
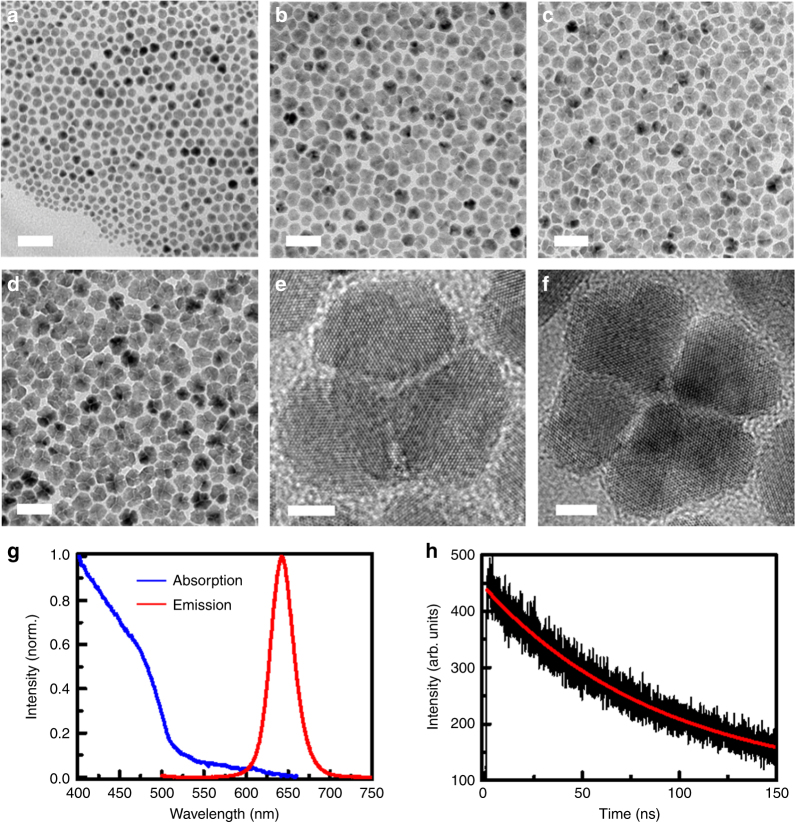
Structural images and ensemble optical properties of gNCCs. **a**,** b**,** c**, **d** TEM (transmission electron microscopy) images showing the formation of CdSe/CdS gNCCs with the CdS shell thicknesses of 5, 10, 15, and 20 MLs, respectively. **e**,** f** High-resolution TEM images of two single CdSe/CdS gNCCs. **g** Absorption and emission spectra of ensemble CdSe/CdS gNCCs. **h** Photoluminescence (PL) decay curve of ensemble CdSe/CdS gNCCs fitted with a single-exponential lifetime of 93.1 ns. The optical measurements in (**g**) and (**h**) were performed at room temperature. The scale bars used in (**a**–**d**) represent 50 nm, while those in (**e**) and (**f**) represent 5 nm

Following a similar procedure as reported previously^12,13^, monodisperse CdSe/CdS gNCs could be synthesized with a CdSe core diameter of 4.2 nm and a CdS shell thickness up to 20 monolayers (MLs) using oleylamine and octadecane as the passivating ligands (see Methods). However, when dioctylamine and 1-octadecence were chosen instead as the passivating ligands and multiple purification procedures were employed (see Methods), the flower-like CdSe/CdS gNCCs started to be formed and reached a stable structure at the CdS thicknesses of 10 and 20 MLs, respectively (see Fig. 1a–d for the transmission electron microscopy (TEM) images). As shown in Fig. 1e, f from the high-resolution TEM images, each gNCC is composed of about 4–5 single gNCs with separation distances of 1–2 nm. At room temperature, the band-edge absorption and emission peaks of ensemble gNCCs are located at 610 nm and 640 nm (Fig. 1g), respectively, while the PL decay curve measured at the emission peak could be roughly fitted with a single-exponential lifetime of 93.1 ns (Fig. 1h).

During the multiple purification processes in the synthesis of gNCCs, it was possible for the passivating ligands of dioctylamine and 1-octadecence to be significantly removed from the NC surface to cause a limited ligand protection (LLP). Mainly due to this LLP condition, three-dimensional assembling of individual gNCs would occur to minimize the total surface free energy, as suggested previously to be responsible for the controllable synthesis of flower-like In_2_O_3_ NC clusters^17,18^. Another driving force for the formation of flower-like gNCCs could be the dipole interaction between neighbouring gNCs, which was successfully manipulated in earlier works to align single spherical CdTe^19^ and ZnO^20^ NCs into one-dimensional nanowires and nanorods, respectively. This point can be partially corroborated by the fact that the visible lattice fringes were found to run across an entire gNCC with their orientation being not disturbed by the visible gaps between neighbouring gNCs (see Fig. 1e, f). Although the exact origin needs to be further investigated, these novel flower-like gNCCs could be reproducibly synthesized in our laboratory to provide a unique platform for the revelation of delicate inter-gNC interactions with a single-particle precision.

### Single-particle optical measurements at room temperature

**Fig. 2:**
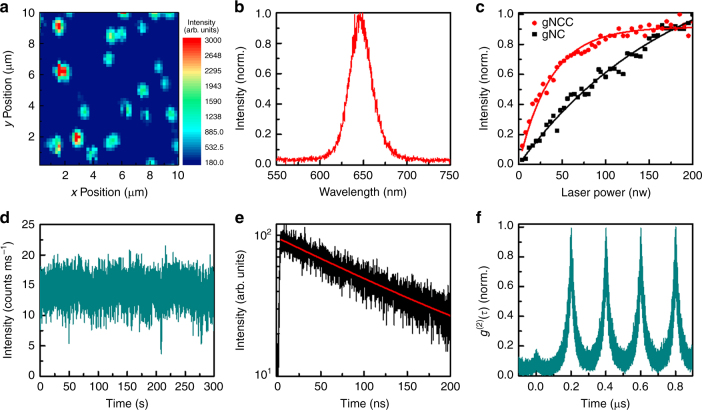
Optical properties of single gNCCs at room temperature. **a** Confocal scanning PL image of single gNCCs. **b** PL spectrum of a single gNCC. **c** PL intensities measured as a function of the laser excitation powers for a single gNCC and a single gNC with the same CdS shell thickness of 20 MLs. These two sets of data points were normalized according to their respective maximum PL intensities obtained within the displayed laser power range. **d** PL intensity vs. time trace measured for a single gNCC with a time resolution of 10 ms. **e** PL decay curve measured for a single gNCC and fitted with a single-exponential lifetime of 138.6 ns. **f** Second-order photon autocorrelation measurement of a single gNCC with a g^(2)^(*τ*) value of about 0.15 at the zero-time delay

At room temperature, single CdSe/CdS core/shell gNCCs with a shell thickness of 20 MLs were excited by a 405 nm pulsed laser (see Methods), whose fluorescence spots can be clearly seen from the confocal scanning PL image shown in Fig. 2a. The PL spectrum of a single gNCC is shown in Fig. 2b with a linewidth of 85.3 meV that is slightly smaller than that of 95.7 meV measured for ensemble gNCCs in Fig. 1g. As shown in Fig. 2c for a representative gNCC, its PL intensity gets saturated at high laser powers due to the nonradiative Auger recombination of multiple excitons^21^. With *j* representing the pump fluence of the excitation laser, this PL saturation curve can be fitted with a functional form, ∝1 − e^−<*N*>^ = 1 − e^−*j*σ^, to yield an absorption cross section (*σ*) of 5.11 × 10^−13^ cm^2^. Once *σ* was determined for a single gNCC, the pump fluence *j* could be adjusted to set < *N* > (the average number of photons absorbed per pulse per gNCC^22,23^) to 0.1 unless otherwise specified in the text. Nearly all of the single gNCCs studied in our experiment presented a nonblinking PL behaviour (Fig. 2d) with a PL decay lifetime ranging from 90–140 ns (Fig. 2e).

The typical value of 5.11 × 10^−13^ cm^2^ for the *σ* of a single gNCC is about four times larger than that of 1.24 × 10^−13^ cm^2^ measured for single gNCs with the same CdS shell thickness of 20 MLs (Fig. 2c). Thus, the early saturation of the PL intensity of a single gNCC with the increasing laser power strongly implies that its composing gNCs must interact efficiently with each other to nonradiatively dissipate their photo-excited excitons. This was further supported by the second-order photon autocorrelation measurements showing that the *g*^(2)^(*τ*) value at the zero-time delay between two consecutively emitted photons is less than 0.15 for all of the single gNCCs studied in our experiment (Fig. 2f). In contrast, the collective fluorescent photons emitted by 4–5 non-interacting quantum emitters should have a *g*^(2)^(*τ*) value greater than 0.75 at the zero-time delay^24^ (see Supplementary Figure 1 for the photon antibunching measurement of several isolated gNCs). The Förster ET process was previously invoked to explain the weak photon antibunching effect observed in single clusters of CdSe/ZnS core/shell NCs, where a *g*^(2)^(*τ*) value greater than 0.7 at the zero-time delay was obtained when more than four individual CdSe/ZnS NCs were assembled together with a separation distance less than 10 nm^8,25^. Given the center-to-center distance of about 20 nm between two nearby CdSe/CdS gNCs in a single gNCC (Fig. 1e, f), the ET efficiency should approach zero according to their ET radius of about 9.1 nm estimated from the standard Förster theory^26^.

### Single-particle optical measurements at 4 K

**Fig. 3:**
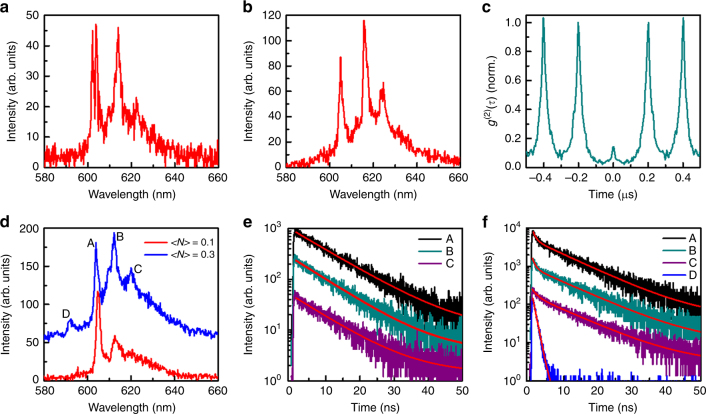
Optical properties of single gNCCs at 4 K. **a**,** b** PL spectra of two single gNCCs excited at < *N* > = 0.1. **c** Second-order photon autocorrelation measurement of a single gNCC with a *g*^(2)^(*τ*) value of about 0.15 at the zero-time delay. **d** PL spectra of a single gNCC excited at < *N* > = 0.1 and 0.3, respectively. **e** PL decay curves measured at < *N* > = 0.1 for the A, B, and C emission peaks of this single gNCC and fitted with single-exponential lifetimes of 10.9, 10.0, and 10.1 ns, respectively. **f** PL decay curves measured at < *N* > = 0.3 for the A, B, and C emission peaks of the same single gNCC. These curves are all fitted by biexponential functions with the fast/slow lifetimes of 0.8/10.1, 0.7/10.5, and 1.1/10.1 ns, respectively. The PL decay curve of an additional emission peak of D is also presented and fitted with a single-exponential lifetime of 0.9 ns

To reveal more details about the optical properties of single gNCCs, we performed additional PL measurements at the cryogenic temperature of 4 K (see Methods). As shown in Fig. 3a, b, all of the studied single gNCCs possessed multiple emission peaks without an obvious relationship between their PL intensities and energies, which provided another evidence that the Förster ET process was not occurring from smaller to larger gNCs within a single gNCC^3,4^. The PL linewidths of the multiple emission peaks from a single gNCC are as broad as 10 meV, which could be caused by the coherent couplings among the interacting gNCs^5–7,10,11^. The energy separation between two nearby PL peaks of a single gNCC varies from 5–55 meV (see Supplementary Figure 2 for a statistical histogram of the energy separations), thus excluding efficient participations of CdS and CdSe LO phonons in the exciton recombination processes since their energies should be fixed at about 27 and 37 meV, respectively^27^ (see Supplementary Figure 3 for the phonon replicas in the PL spectra of two individual gNCs). Collective fluorescent photons from these multiple emission peaks still demonstrated a strong photon antibunching effect, with a *g*^(2)^(*τ*) value also less than 0.15 at the zero-time delay for all of the studied single gNCCs (Fig. 3c).

Based on the above experimental results, we tentatively propose that it is the inter-gNC Auger interaction that leads to the high-purity single-photon emission from a single gNCC. For the simplest scenario with two photo-excited gNCs, the exciton in one gNC can transfer its recombination energy nonradiatively to the exciton in the other gNC, which jumps to a higher excited state and then relaxes back to emit a photon. The possible existence of this novel type of Auger interaction can be deduced from the space-separated quantum cutting (SSQC) effect extensively studied in solid-state dispersions of Si NCs in a SiO_2_ matrix^28–31^. In this Auger-type SSQC process, a hot carrier excited in a single Si NC can decay directly to the band-edge state with the released energy creating an electron-hole pair in another Si NC or an excited-state electron in an Er^3+^ ion located within a nanometer-scale distance. Meanwhile, it is also likely for the excited-state electron of the Er^3+^ ion to transfer its energy back to the carrier of the original Si NC by a reverse Auger process^28,32^, which is similar to what we are proposing here as the origin of the exciton–exciton annihilation interaction between nearby gNCs. The Förster ET process relies on the center-to-center distance of the donor and acceptor emitters, while the SSQC and its reverse processes are dependent on the edge-to-edge distance that is critical for the overlap of their electronic wave functions from a quantum point of view^33^.

In fact, the inter-gNC Auger interaction proposed here is also reminiscent of the singlet–singlet annihilation (SSA) phenomenon manifested in single multichromophoric molecules^34^. When SSA occurs, the recombination energy of one singlet exciton in a chromophore could be transferred to the singlet exciton in another chromophore separated by an inter-chromophore distance as large as several nanometers, which would eventually yield a *g*^(2)^(*τ*) value less than 0.1 at the zero-time delay after the remaining exciton relaxes back from the higher singlet state to emit a photon^24,35^. It was suggested that the SSA should be either realized by^24,36^ or compete with^37,38^ the incoherent Förster-type ET process, and in the latter case it might involve coherent delocalization of the singlet-exciton wave functions^24^. As mentioned earlier in the text^5–7^, strong electronic coupling between neighbouring colloidal NCs in a solid film can be achieved due to the extension of carrier wave functions across a distance gap of several nanometers, which would prevent dipole formation at each NC center to suppress the long-range Förster ET^39^. Specifically in the CdSe/CdS gNCs, it has been widely confirmed that the wave function of photo-excited electrons is spatially delocalized over the whole core/shell structure^27,40^ so that coherent coupling between nearby gNCs in a single gNCC could be reasonably expected.

For a better understanding of the inter-gNC Auger recombination process, we performed the PL spectral and decay measurements of single gNCCs as a function of the laser excitation power also at the cryogenic temperature of 4 K. In Fig. 3d, we plot the PL spectra of a representative gNCC excited at both < *N* > = 0.1 and 0.3, where the same multiple emission peaks marked by A, B, and C should arise from single-exciton recombination of different gNCs (for comparison, see Supplementary Figure 4 for the PL spectra of an individual gNC measured at different laser powers). At low-power excitation when at most one photon from a laser pulse is absorbed by the gNCC, any of the composing gNCs could be excited with a specific probability so that multiple emission peaks could be acquired after many pulses of laser excitation. Due to the size-dependent absorption cross section, the probability of absorbing a photon should be higher for the larger gNC emitting with a longer wavelength within the cluster, which is not reflected in Fig. 3a, b from the multiple emission peaks of single gNCCs excited with a low laser power. In addition to the absorption cross section, there might exist other factors to influence the relative PL intensities of the composing gNCs within a cluster, such as the detailed electronic structure at the excitation wavelength, the orientation of the absorbing/emitting dipole moment and the fluorescent quantum yield. When the laser power is further increased with all the other factors being unchanged, the absorption cross section might play a dominant role in increasing the relative PL intensities of the larger-sized gNCs in the cluster (see Fig. 3d). With a high laser power to excite multiple gNCs in the cluster, we tentatively assume that there is no preference for the largest or smallest gNC to have a survival exciton after the inter-gNC Auger interaction process. This is similar to the case with two perylenemonoimide chromophores both in their first excited singlet states, where the SSA process was proposed to occur in two directions^41^.

As shown in Fig. 3e, the PL decay curves measured at < *N* > = 0.1 for the A, B, and C peaks can all be fitted by single-exponential functions with similar lifetimes of 10.9, 10.0, and 10.1 ns, respectively, to verify again that the Förster ET process could be neglected. With the laser excitation at < *N* > = 0.3, the PL decay curves shown in Fig. 3f for the A, B, and C peaks can only be fitted by biexponential functions with the fast/slow lifetime components of 0.8/10.1, 0.7/10.5, and 1.1/10.1 ns, respectively. For the PL spectrum obtained at < *N* > = 0.3 in Fig. 3d, there appears an additional emission peak of D whose PL decay curve shown in Fig. 3f can be well fitted with a single-exponential lifetime of 0.9 ns. We speculate that the D peak should be related to the biexciton emission since its energy is about 40 meV higher than that of the A peak, which is close to the average value of 25 ± 5 meV previously measured between the biexciton and single-exciton emission peaks of individual gNCs due to the strong exciton–exciton repulsion effect^16^ (also see Supplementary Figure 4(f) for the biexciton recombination from an individual gNC). Due to the weak PL signal and the extremely fast PL lifetime, the biexciton D peak should be dominated by the nonradiative Auger recombination. Within the emission energy range of the A, B, and C peaks in Fig. 3d ( < *N* > = 0.3), there might exist other biexciton emission peaks to yield their respective fast lifetime components in Fig. 3f. So far, it is hard to judge whether the biexciton peak originates from a single gNC or involves different gNCs in the gNCC, but its PL lifetime around 1.0 ns has at least provided a coarse estimation of the time window for the inter-gNC Auger interaction process to occur. Finally, It should be noted that the *g*^(2)^(*τ*) value measured at the zero-time delay for a single gNCC increases slightly with the laser excitation power (see Supplementary Figure 5), which should be caused by the contribution of more fluorescent photons from biexcitons and higher-order multi-excitons^42,43^.

## Discussion

To summarize, we have demonstrated that several single CdSe/CdS gNCs can be assembled together to form a flower-like gNCC capable of emitting high-purity single photons. This strong photon antibunching effect is attributed to the inter-gNC Auger interaction process that can nonradiatively dissipate photo-excited excitons located at different gNCs so that only one single exciton is eventually left to emit a photon. Fundamentally, the preparation of such a gNCC system has provided an unprecedented opportunity for the investigation of how the optoelectronic properties evolve from a single NC to the NC molecule. Technically, the suggested mechanism of inter-gNC Auger interaction would have profound influences on a variety of optoelectronic devices whose operation parameters are strongly dependent on the mutual interactions of closely packed gNCs. We believe that the observation of this inter-gNC Auger interaction scheme depends critically on the large center-to-center distance of neighbouring gNCs that minimizes the Förster ET influence, as well as the great wave-function extension of the photo-excited electrons to the CdS shell that maximizes the coherent coupling effect. However, more theoretical treatments and experimental measurements are still needed in future works to determine whether this novel mechanism is only limited to the CdSe/CdS gNCs or can be generally extended to other colloidal NCs.

## Methods

### Chemicals

Cadmium oxide (99.99%), sulfur powder (99.98%), and oleylamine (70%) were purchased from Aldrich. Selenium powder (200 mesh, 99.999%), 1-octadecence (90%), octadecylamine (97%), dioctylamine (96%), oleic acid (90%), and octadecane (90%) were purchased from Alfa-Aesar. Tributylphosphine (95%) was purchased from Aladdin. Hexane, toluene, aceton, and methanol were purchased from Sinopharm Chemical Reagents. All chemicals were used directly without any further purification.

### Precursor preparation

The selenium precursor solution (2.4 M) was prepared by dissolving selenium powder (1.9 g) in tributylphosphine (10 mL) at room temperature. The sulfur precursor solution (0.1 M) was prepared by dissolving sulfur powder (0.0962 g) in 1-octadecence at 100 °C. To prepare the cadmium oleate precursor solution (0.1 M), cadmium oxide (0.385 g), oleic acid (6.779 g), and 1-octadecence (17.64 g) were mixed in a 100 mL three-neck flask. After degassing for 20 min, the mixture in the flask was heated to 250 °C to get a clear solution. This precursor solution was allowed to cool down to 40 °C and then transferred to a 40 mL glass bottle. All of the above precursor solutions were made and stored under an argon atmosphere.

### Synthesis of CdSe core NCs

After degassing for 20 min, the mixture of cadmium oleate precursor solution (2 mL), octadecylamine (2 g), and 1-octadecence (4 mL) in a 25 mL three-neck flask was heated to 280 °C under argon flow. The selenium precursor solution (0.5 mL) was quickly injected and the growth temperature was then reduced to 250 °C. After the growth of suitable sizes, the heating mantle was removed and the reaction mixture was allowed to cool down to room temperature. After a centrifugal separation procedure for the purification, the reaction mixture was added into 40 mL of acetone and then centrifuged at 4000 rpm for 10 min. The final product of CdSe NCs was redispersed in hexane as the core stock solution.

### Synthesis of flower-like CdSe/CdS gNCCs

The particle concentration of the CdSe core stock solution in hexane was measured using Beer’s law with the extinction coefficient of CdSe NCs. The amount of cadmium or sulfur precursor was determined by the molar weight of the atoms required by the volume increment of each CdS ML with a thickness of 0.35 nm. The CdSe core stock solution was mixed with 2 mL of dioctylamine and 12 mL of 1-octadecence in a 100 mL three-neck flask, which was then pumped under vacuum for 20 min at 60 °C to remove the hexane. Subsequently, the system was switched to the argon flow and the reaction mixture was further heated to 240 °C. Based on the successive ion layer adsorption and reaction (SILAR) approach with small modifications, the cadmium and sulfur precursors were injected alternately into the above solution containing CdSe core NCs. After 20 min for the deposition of 5 MLs of the CdS shell, the reaction was stopped and the solution was cooled down to room temperature for the centrifugal separation purification. The above synthesis procedure was repeated three more times to obtain the CdS shell thicknesses of 10, 15, and 20 MLs with the reaction times of 60, 90, and 180 min, respectively, while the reaction temperature was always kept at 240 °C.

### Synthesis of monodisperse CdSe/CdS gNCs

The particle concentration of the CdSe core stock solution in hexane was estimated as above. The amount of cadmium or sulfur precursor was also determined by the molar weight of the atoms required by the volume increment of each ML of the CdS shell. The CdSe core stock solution was mixed with 2 mL of oleylamine and 6 g of octadecane in a 100 mL three-neck flask, which was then pumped under vacuum for 20 min at 60 °C to remove the hexane. Subsequently, the system was switched to argon flow and the reaction mixture was further heated to 240 °C for the alternate injections of cadmium and sulphur precursors. After 20 min for the deposition of 5 MLs of the CdS shell, the reaction was stopped and the solution was cooled down to room temperature without any further purification. The above synthesis procedure was repeated three more times to obtain the CdS shell thicknesses of 10, 15, and 20 MLs with the reaction times of 40, 70 and 90 min, and the reaction temperatures of 280, 240, and 240 °C, respectively. The final product of CdSe/CdS gNCs with a CdS shell thickness of 20 MLs was purified by centrifugation and redispersed in toluene to form a long-term stable solution.

### Optical measurements

One drop of the concentrated or diluted solution of colloidal NCs (CdSe/CdS gNCs or gNCCs) was spin-coated onto a fused silica substrate to form a solid film for the ensemble or single-particle optical characterizations at room temperature. The 405 nm output of a picosecond diode laser operated at a repetition rate of 5 MHz was focused onto the sample substrate by an immersion-oil objective (N.A. = 1.4). PL signal of the ensemble NCs or a single NC was collected by the same objective and sent through a 0.5 m spectrometer to a charge-coupled-device camera or an avalanche photo diode (APD) for the PL spectral measurement with an integration time of 1 s or the PL decay measurement with a time resolution of about 250 ps. The PL signal of a single NC can be alternatively sent through a non-polarizing 50/50 beamsplitter to two APDs for the second-order photon autocorrelation measurement. For the low-temperature optical characterizations of single NCs, the sample substrate was contained in a helium-free cryostat operated at 4 K. Very similar optical setups and measurements to those described above were employed except that the immersion-oil objective was replaced by a dry objective (N.A. = 0.8).

### Data availability

The data supporting the findings of this study are available from the corresponding authors upon request.

## Electronic supplementary material

Supplementary Information
