# Supplementary material for: Photon antibunching in a cluster of giant CdSe/CdS nanocrystals
Source: Nat Commun. 2018 Apr 18;9:1536. doi: 10.1038/s41467-018-03971-w (PMC5906464; doi:10.1038/s41467-018-03971-w)
Supplement: Supplementary file 1 — Supplementary Information [file 41467_2018_3971_MOESM1_ESM.pdf]

Photon Antibunching in a Cluster of Giant CdSe/CdS Nanocrystals

Bihu Lv,[1†] Huichao Zhang,[1,3†] Lipeng Wang,[1] Chunfeng Zhang,[1] Xiaoyong Wang,[1*] Jiayu Zhang,[2*] and Min Xiao[1,4*]

[1]*National Laboratory of Solid State Microstructures, School of Physics, and Collaborative Innovation Center of Advanced Microstructures, Nanjing University, Nanjing 210093, China*

[2]*Advanced Photonics Center, School of Electronic Science and Engineering, Southeast University, Nanjing 210096, China*

[3]*College of Electronics and Information, Hangzhou Dianzi University, Xiasha Campus, Hangzhou 310018, China*

[4]*Department of Physics, University of Arkansas, Fayetteville, Arkansas 72701, USA*

[†]These authors contributed equally to this work.

[*]Correspondence to X.W. (wxiaoyong@nju.edu.cn), J.Z. (jyzhang@seu.edu.cn), or M.X. (mxiao@uark.edu).

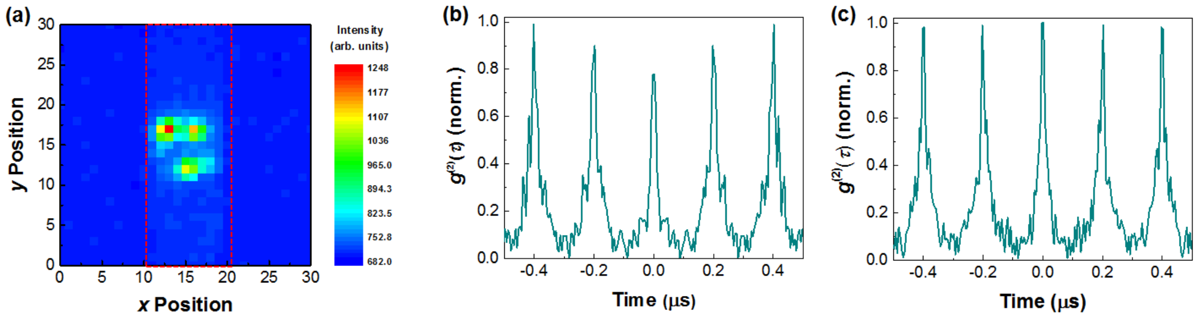

Supplementary Figure 1 | Photon antibunching measurements of several isolated gNCs. (a) Photoluminescence (PL) image of three isolated CdSe/CdS gNCs excited at $<N> = 0.1$ with an acquisition time of 1 s. The red dotted box marks the profile of the spectrometer entrance slit corresponding to a sample region of 3.2 μm × 9.6 μm. **(b)** Second-order photon autocorrelation measurement of these three gNCs with a $g^{(2)}(\tau)$ value of 0.78 at the zero-time delay. **(c)** Second-order photon autocorrelation measurement of >5 gNCs with a $g^{(2)}(\tau)$ value approaching one at the zero-time delay. The above optical measurements were performed at the cryogenic temperature of 4 K.

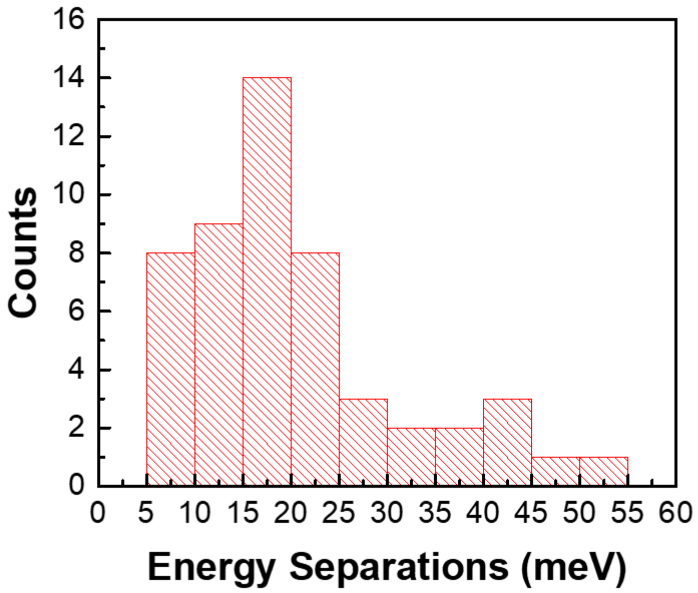

Supplementary Figure 2 | Energy separations of nearby PL peaks in single gNCCs. Statistical histogram for the energy separations between all the nearby peaks in the PL spectra of >30 single gNCCs excited at 4 K with <*N*> = 0.1.

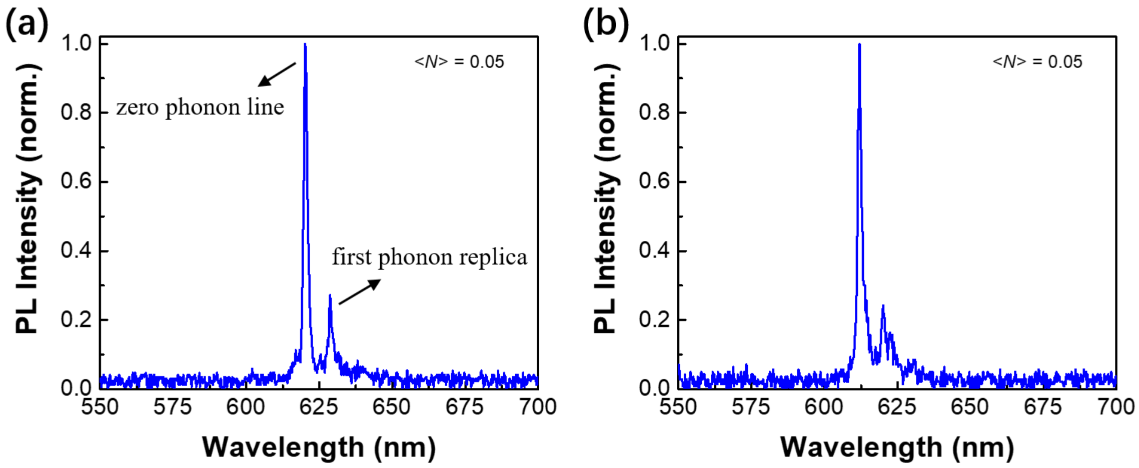

Supplementary Figure 3 | PL spectra of individual gNCs measured at 4 K. (a), (b) PL spectra measured with an integration time of 1 s for two representative gNCs excited at <*N*> = 0.05 with the energy separations of 27.5 and 26.5 meV, respectively, between the zero phonon line and the first phonon replica.

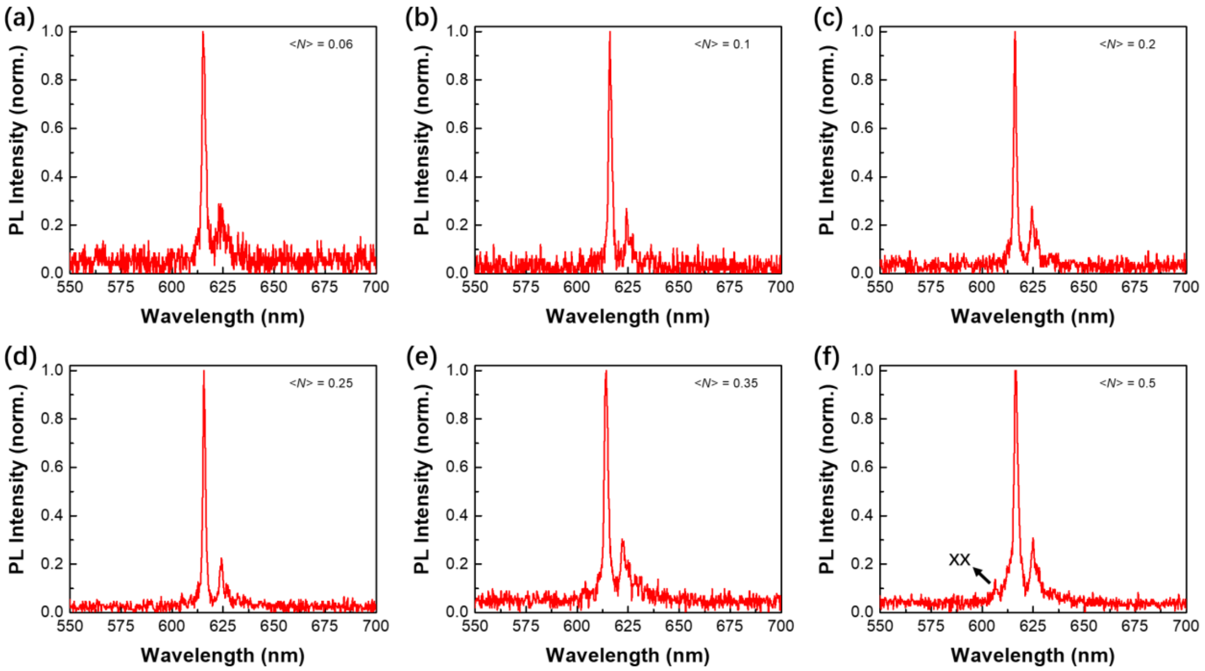

Supplementary Figure 4 | PL spectra of an individual gNC measured at 4 K. PL spectra measured with an integration time of 1 s for a representative gNC excited at **(a)** <*N*> = 0.06, **(b)** <*N*> = 0.1, **(c)** <*N*> = 0.2, **(d)** <*N*> = 0.25, **(e)** <*N*> = 0.35, and **(f)** <*N*> = 0.5, respectively. It should be noted that a very weak peak appears on the blue side of the PL spectrum measured at <*N*> = 0.5 and is attributed to the biexciton (XX) recombination with an emission energy 32.1 meV higher than that of the main peak from single excitons.

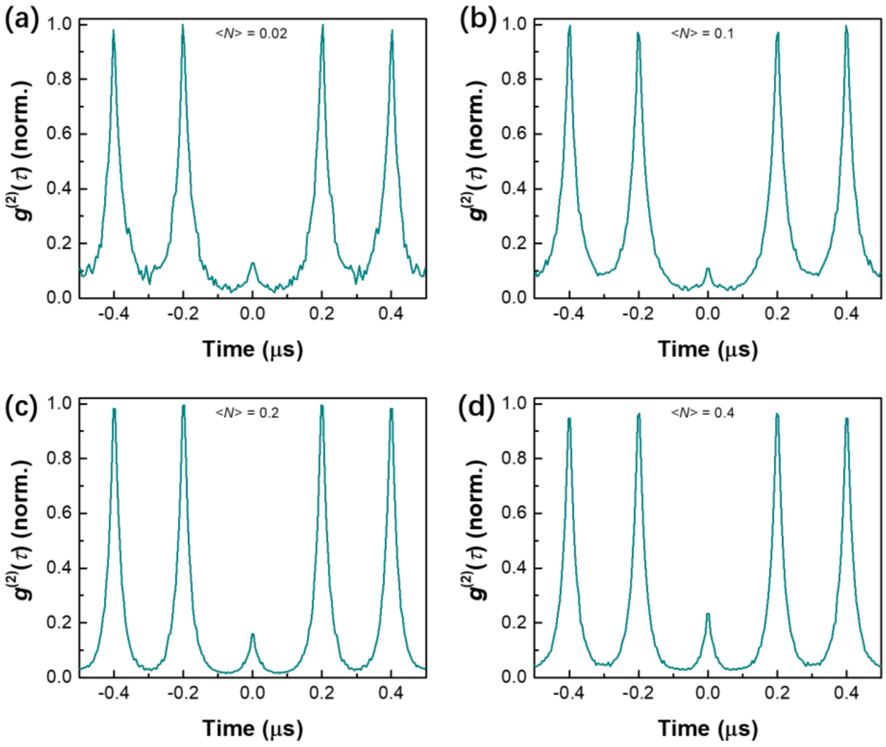

Supplementary Figure 5 | Photon antibunching of an individual gNCC measured at 4 K.

The laser excitation power corresponds to **(a)** <*N*> = 0.02, **(b)** <*N*> = 0.1, **(c)** <*N*> = 0.2, and **(d)** <*N*> = 0.4, respectively.
